# Total and live birth prevalence of singleton pregnancies with Down's syndrome in Scotland between 2000 and 2021: a population-based study

**DOI:** 10.1016/j.lanepe.2026.101639

**Published:** 2026-03-06

**Authors:** Elinor Sebire, Rachael Wood, Clara Calvert, Rute Vieira

**Affiliations:** a Institute of Applied Health Sciences, Polwarth Building, University of Aberdeen, Aberdeen, Scotland; b Public Health Scotland, Edinburgh, Scotland

**Keywords:** Down's syndrome, Prevalence, Temporal trend, Socio-demographic factors, Scotland

## Abstract

**Background:**

Down's syndrome (DS) is the most common chromosomal congenital condition diagnosed in pregnancy. Antenatal screening for DS is available in Scotland, and in September 2020, non-invasive prenatal testing (NIPT) was added to the screening pathway. This study aimed to examine trends in total birth (TB) and live birth (LB) prevalence of DS (2000–2021) in Scotland and associations with maternal and infant socio-demographic factors.

**Methods:**

A retrospective, observational, population-based study using the Scottish Linked Congenital Conditions Dataset (SLiCCD). Poisson and generalised Poisson regression analyses were used to model TB and LB prevalences of DS. Associations with year, maternal age, Scottish Index of Multiple Deprivation, health board of residence, and infant sex (live births only) were assessed. Prevalence rate ratios (PRR), 95% confidence intervals (CIs) and p values were reported.

**Findings:**

There were 2098 singleton pregnancies and 1135 live births with DS (2000–2021), with a TB prevalence of 17.4 (95% CI 16.7, 18.2) per 10,000 TBs, and LB prevalence of 9.5 (95% CI 8.9, 10.0) per 10,000 LBs. There was evidence of a non-linear time trend in TB prevalence, increasing between 2013 and 2015 [PRR: 1.13 (95% CI 1.07, 1.19); p < 0.001] and decreasing between 2016 and 2021 [PRR: 0.95 (95% CI 0.92, 0.98); p = 0.002]. No time trend was observed for LB prevalence. Maternal age and health board of residence were associated with TB and LB prevalence of DS.

**Interpretation:**

Our population-based insights into TB and LB prevalence of DS in Scotland are important in establishing baseline trends to investigate the impact of NIPT. This enables up-to-date counselling for those undergoing DS screening, and appropriate planning of healthcare services.

**Funding:**

ES received 10.13039/501100000294Medical Research Scotland PhD Studentship funding (grant no. PHD-50200-2020).

**Research in context**

**Evidence before this study**

Biochemical screening for Down's syndrome (DS) during pregnancy has been available in the UK since the 1990s, but it was not coordinated nationally until 2001, when guidelines on the offer of DS screening were published by the UK National Screening Committee. There have been several changes to the antenatal screening pathway since then, with the most recent being the introduction of non-invasive prenatal testing (NIPT) into the Scottish antenatal screening pathway for DS in September 2020. This prompted an evaluation of the impact of implementing this new test on screening decisions and pregnancy outcomes following a diagnosis of DS. To achieve this, pre-implementation secular trends in total birth and live birth prevalence of DS in Scotland must first be understood, as well as maternal and infant sociodemographic factors that may be associated with prevalence.

Public Health Scotland's report Congenital conditions in Scotland, 2000–2020 reported annual total birth and live birth prevalence of DS in Scotland. However, DS-specific time trends in prevalence and their associations with maternal and infant sociodemographic factors are not currently available for the Scottish population.

**Added value of this study**

This study examines changes in total birth and live birth prevalence of DS in Scotland between 2000 and 2021, and investigates associations with maternal and infant sociodemographic factors.

**Implications of all the available evidence**

In line with DS prevalence observed in other populations, the results confirm a strong association between both total birth and live birth prevalence of DS and maternal age, and also demonstrate differences in prevalence by NHS health board of residence. The findings establish baseline trends to monitor the impact of NIPT in Scotland and provide detailed information on singleton pregnancies in which the baby was diagnosed with DS between 2000 and 2021, to inform counselling for women undergoing screening. This population-based research can also support appropriate planning of public health programmes and service delivery.

## Introduction

Down's syndrome (DS) is a genetic condition caused by an additional copy of chromosome 21 and is the most commonly occurring chromosomal condition in humans.^1^^,^^2^ It is associated with learning disability and an increased risk of Alzheimer's, congenital heart, metabolic, and autoimmune diseases.^2^, ^3^, ^4^, ^5^, ^6^ DS is also associated with a decreased risk of some cancers, such as breast cancer, and of atherosclerosis.^1^ Advances in medical and social care for people with DS have led to improved quality of life and increased life expectancy to 60–70 years of age.^4^

DS can be identified during pregnancy, allowing appropriate planning and health monitoring. In Scotland, pregnancy screening tests for DS were first nationally coordinated in 2001, following recommendations by the UK National Screening Committee and development of the maternity services framework.^7^^,^^8^ This framework was implemented to ensure screening offered by Scottish National Health Service (NHS) health boards was consistent.^9^^,^^10^ Currently, first-line (combined or quadruple) screening is offered to all pregnant women booking for antenatal care in Scotland. Combined screening includes a first-trimester ultrasound dating scan, nuchal translucency measurement and blood serum screening,^8^^,^^11^ while quadruple screening is a second-trimester blood serum test. Those with a higher-chance first-line screening result are offered non-invasive prenatal testing (NIPT) or an invasive prenatal diagnostic (IPD) test.^11^^,^^12^ The screening pathway for DS in Scotland has undergone various changes since 2001, most recently the introduction of NIPT in September 2020.^11^^,^^13^ Before this, higher-chance pregnancies for DS were offered IPD testing only. As such, NIPT (second-line screening) provides an additional and more accurate screening option for women with a higher-chance first-line screening result.

It is important to understand the impact of implementing screening pathway changes, such as NIPT, which may affect the timing of and decisions regarding screening or further diagnostic testing, and the likelihood of identifying babies with DS. More babies diagnosed prior to a spontaneous pregnancy loss or termination of pregnancy (TOP) could influence the total birth (TB) prevalence of DS in the population. Baseline data on long-term trends in TB and live birth (LB) prevalence of DS in Scotland can help explore the potential impact of changes to the screening pathway for DS.

Between 1995 and 2015, the LB prevalence of DS in Scotland was 9.8 per 10,000 LBs.^14^ The most recent official statistics reported by Public Health Scotland (PHS) showed a TB prevalence of DS in Scotland of 18.4 per 10,000 TBs and a LB prevalence of 7.8 per 10,000 LBs in 2020.^15^ Among other countries submitting data to the *European network of population-based registries for the epidemiological surveillance of congenital anomalies* (EUROCAT), the overall LB prevalence of DS was 9.90 (95% CI 9.74, 10.06) per 10,000 LBs between 2000 and 2021.^16^ Variation in prevalence estimates between populations and over time reflects multiple factors–not only how prevalence is defined and calculated, and the data sources used, but also the socio-demographic makeup of a population, access to healthcare, and availability of pregnancy screening services, which may affect the likelihood of identifying a pregnancy with DS. Despite the availability of data and reported prevalence estimates, no comprehensive or comparable analyses investigating changes in DS prevalence over time, and associations with socio-demographic factors have been reported for Scotland.

Real-world, population-level data are essential for a national health service that meets population needs. Data on TB prevalence of DS and its associations with socio-demographic factors are important for understanding an individual's baseline chance of having a baby with DS and may influence screening or diagnostic testing decisions. Trends in LB prevalence and socio-demographic associations are also important for resource allocation and delivery of appropriate healthcare services. This can help better support children with DS, who are more likely to be hospitalised and readmitted than their peers.^14^

Using the Scottish Linked Congenital Conditions Dataset (SLiCCD), this study aimed to investigate trends over time in TB and LB prevalence of DS in Scotland, and associations with maternal and infant socio-demographic factors between 2000 and 2021. Reporting these long-term, population-based trends is important for fundamental knowledge of the condition,^5^ for providing evidence-based information during pregnancy screening, and for establishing baseline data to monitor the impact of screening pathway changes.

## Methods

This retrospective, population-based study followed the STROBE reporting guidelines for observational studies.^17^ The study protocol was written *a priori* and is available online at: https://www.abdn.ac.uk/iahs/research/specialist-collaborations/nipt-downs-syndrome/#panel65386 (URL).

### Data sources and procedures

#### Access and permissions

All data extracts were provided by PHS and were kept and analysed by researchers within PHS's secure servers. Permissions for accessing and analysing this data were granted through the Public Benefit and Privacy Panel for Health and Social Care (HSC-PBPP) agreement (see section 2.4). All data were pseudo-anonymised by PHS analysts and anonymised to researchers before access. This research followed the PHS statistical disclosure control guidance for all outputs.^18^

#### Inclusion criteria

Singleton pregnancies in Scotland ending between 1 January 2000 and 31 December 2021, where the baby was diagnosed with DS at any point during pregnancy and up to one year after birth.

#### Scottish Linked Congenital Conditions Dataset (SLiCCD)

SLiCCD was created by linking existing national health records held by PHS, including National Records Scotland (NRS) and Scottish Morbidity Records (SMR), and covers pregnancies ending in Scotland in 2000–2021. National data sources were linked using the maternal community health index (CHI) number, a unique identifier, and pregnancy end date, to identify pregnancies and additional clinical and socio-demographic information.^19^ See the Appendix for the full list of data sources used in SLiCCD.

Pregnancies with a recognised condition or syndrome were included if the baby was live born and diagnosed within the first year of life; spontaneous stillbirth (≥24 weeks gestation); spontaneous foetal loss (20–23 weeks’ gestation); or TOP at any gestation. Diagnostic codes, based on the International Classification of Diseases, tenth revision (ICD10), were used to identify pregnancies with an included congenital condition and assign them to specific groups defined by EUROCAT.^20^

This study used an extract of SLiCCD including only data on singleton pregnancies with a diagnosis of DS ending in Scotland in 2000–2021. The data variables for each included pregnancy were pregnancy outcome (LB; spontaneous stillbirth; spontaneous foetal loss; TOP), maternal age at conception (<20, 20–24, 25–34, 35–39, 40+, unknown), maternal Scottish Index of Multiple Deprivation (SIMD) quintile at end of pregnancy (1 (most deprived); 2; 3; 4; 5 (least deprived); unknown), maternal NHS health board of residence at end of pregnancy (NHS Ayrshire and Arran (A&A); NHS Borders; NHS Dumfries and Galloway; NHS Fife; NHS Forth Valley; NHS Grampian; NHS Greater Glasgow and Clyde; NHS Highland; NHS Lanarkshire; NHS Lothian; NHS Orkney; NHS Shetland; NHS Tayside; NHS Western Isles; unknown), infant sex (male; female; unknown), maternal ethnicity (African, Caribbean or Black; Asian; Asian Scottish or Asian British; Mixed/multiple ethnic groups and other ethnic groups (including Arab); White; Unknown).

SIMD is the Scottish Government's standard tool for measuring deprivation in Scotland, calculated for each area based on seven domains (income; employment; health; education, skills and training; geographic access to services; crime; housing). It assigns each domain a rank and calculates an overall SIMD score, which is the weighted sum of the seven domain scores for the area.^21^

See the Appendix for more detail on SIMD, and NHS Scotland health boards (Appendix 1, Figure S1, and Table S1).

#### Denominator data for prevalences

In Scotland, only LBs (at any gestation) and stillbirths **≥**24^+0^ weeks’ gestation are registerable by law. Therefore, and in line with PHS official statistics and EUROCAT guidelines,^22^ only stillbirths and LBs registered by NRS were used as the denominator for TB prevalence of DS, while pregnancies with DS ending in spontaneous foetal loss or TOP were included in the numerator of total affected births. LBs in Scotland registered by NRS were used as the denominator for LB prevalence.

Aggregate denominator data were also obtained from NRS, broken down for each year from 2000 to 2021 for singleton TBs (live and stillbirths) and LBs, and by maternal age group, SIMD, NHS health board of residence and, for LBs only, infant sex. Only the LB prevalence was reported by infant sex as sex is often unknown for pregnancies with DS ending in spontaneous foetal loss or TOP.

#### Enhanced SLiCCD (eSLiCCD)

eSLiCCD includes pregnancies ending between 1 April 2019 and 31 December 2020 with a DS, Edwards' syndrome or Patau's syndrome diagnosis sourced from a combination of SLiCCD and genetic testing data. Genetic testing data (provided to PHS by the four NHS clinical genetics laboratories in Scotland) provide the results of any genetic diagnostic tests performed following IPD, post-natally before the first birthday in a live born baby or following pregnancy loss. Genetic testing data were used to confirm or update specific diagnoses, or to add missing records in SLiCCD. Babies within eSLiCCD will therefore have a ‘confirmed’ diagnosis if the initial SLiCCD record matches a genetic testing record or when they have a genetic record only.

### Statistical analysis

Relevant outcomes and factors in the SLiCCD cohort were summarised using frequencies and percentages, including missing data. Multiple imputation of missing data was considered if more than 10% of individuals had missing data in at least one variable in the final multivariable models.

Maternal age was categorised into groups (<25, 25–29, 30–34, 35–39, 40+) and smaller NHS health boards (Western Isles, Shetland, and Orkney) were grouped together as ‘NHS Island Boards’, to avoid disclosing small numbers. The regression analyses reference groups were <25 years for maternal age, quintile 1 for SIMD, and NHS A&A for health board of residence as it had an average population size and no extreme values for DS prevalence per year compared to other health boards.

#### Prevalence of Down's syndrome

The total number of singleton pregnancies with DS ending between January 2000 and December 2021, by pregnancy outcome, the TB prevalence of DS per 10,000 TBs, and the LB prevalence of DS per 10,000 LBs (with 95% confidence intervals (CIs)), were reported (overall and per year).

The prevalence rate ratios (PRRs) describing the time trends of DS TB prevalence (per 10,000 TBs) and LB prevalence (per 10,000 LBs), using yearly time points, between 2000 and 2021, and their respective 95% CIs and p values were estimated using Poisson or, if data were not equidispersed, Conway–Maxwell Poisson (COM-Poisson) regression models.^23^ A linear time trend and a non-linear time trend model with restricted cubic splines were compared. Non-linear trend models were optimised for best fit by adjusting the number and position of the knots to reflect changes observed through visual analysis, with a maximum of four knots being fitted. The Akaike Information Criterion (AIC),^24^ which estimates the relative information lost by a given model, was used to decide on the most representative model. The model with the lowest AIC, by at least two units, was chosen—if the model AICs were less than two units apart, then the simplest model was chosen (i.e. linear or with the fewest knots).^25^

When the yearly time trend was linear, the PRR (95% CI, p value) for the annual change in DS TB and LB prevalence between 2000 and 2021 was reported. For non-linear time trends, different sequential time periods between 2000 and 2021 with a linear time trend were identified, based on the knots used to estimate the non-linear time trend, and piecewise regression was used to quantify the annual change in prevalence within each period. The PRR (95% CI, p value) was then estimated to describe and assess the linear time trends during each period.

All statistical analyses were conducted using R software version 4.1.2. Packages *glmmTMB* and *VGAM* were used for COM-Poisson and Poisson regression, respectively. Code is available at: https://github.com/Public-Health-Scotland/PhD_NIPT_repository_public_ (URL).

#### Association between socio-demographic factors and TB and LB prevalence of DS

TB and LB prevalences of DS between 2000 and 2021 (with 95% CI) were estimated for each socio-demographic factor.

The statistical association between each socio-demographic factor and prevalence was assessed in three stages. Firstly, univariable Poisson regression models estimated the unadjusted PRRs for each socio-demographic factor, separately. Secondly, COM-Poisson regression models estimated PRRs for each socio-demographic factor separately adjusting for the time trends identified in the time trend analyses. Thirdly, the final multivariable models were built by including all socio-demographic factors. PRRs (95% CI, p value) were reported for all models.

Multicollinearity between independent variables was assessed using variance inflation factors (VIF). The association between maternal age and SIMD was also examined using a Cochran-Mantel-Haenszel test (chi-squared test for ordered categorical variables).

#### Validation of SLiCCD against the eSLiCCD dataset

Data from April 2019 to December 2020 were used to calculate the positive predictive value (PPV) and sensitivity of SLiCCD in recording singleton pregnancies with DS compared to eSLiCCD. Pregnancies ending before 20 weeks' gestation, and those with an ‘unknown’ outcome were excluded from eSLiCCD prior to analysis. PPV was the percentage of pregnancies with DS in SLiCCD that were also recorded with DS in eSLiCCD. Sensitivity was the percentage of pregnancies with DS in eSLiCCD that were also recorded with DS in SLiCCD. PPV and sensitivity were also calculated by pregnancy outcome: TOP, stillbirth, and LB.

### Ethics approval

Approval for accessing and analysing the datasets in this research was granted through HSC-PBPP approval (2122-0261), along with an approved PHS Data Protection Impact Assessment (DPIA) for this programme of work (DP21220627).

### Role of the funding source

The funder of this study had no role in study design, data collection, data analysis, data interpretation, or writing of this report.

## Results

There were 2098 singleton pregnancies with a diagnosis of DS in Scotland between 2000 and 2021 (Table 1). The mean maternal age at conception was 34.15 years and almost 80% of the mothers were 30 years old or older. Over half resided in NHS Lothian (20.2% (424/2098)), NHS Greater Glasgow and Clyde (17.8% (374/2098)) and NHS Grampian (13.4% (282/2098)) health boards. Most variables had less than 5% missing/unknown data, except infant sex (38.1% (799/2098)) and maternal ethnicity (58.3% (1224/2098)). Ethnicity was excluded from all further analyses, while infant sex was only included in LB analyses as it was assigned to all LBs. The characteristics of pregnancies ending between 2000 and 2019 (pre-NIPT implementation) are in Appendix 1, Table S2.

**Table 1:** Baseline characteristics.

	Singleton pregnancies of babies diagnosed with Down's syndrome (n = 2098)
Pregnancy outcome	
Live births	1135 (54.1%)
Stillbirths	78 (3.7%)
Spontaneous foetal loss	4 (0.2%)
Termination of pregnancy	881 (42.0%)
Maternal age (grouped years, at conception)	
<20	67 (3.2%)
20–24	123 (5.9%)
25–29	241 (11.5%)
30–34	491 (23.4%)
35–39	761 (36.3%)
40+	408 (19.5%)
Unknown	7 (0.3%)
Maternal age (years, at conception)	
Mean (SD)	34.15 (6.29)
NHS health board of residence	
NHS Ayrshire and Arran	131 (6.2%)
NHS Borders	21 (1.0%)
NHS Dumfries and Galloway	54 (2.6%)
NHS Fife	154 (7.3%)
NHS Forth Valley	112 (5.3%)
NHS Grampian	282 (13.4%)
NHS Greater Glasgow and Clyde	374 (17.8%)
NHS Highland	142 (6.8%)
NHS Lanarkshire	207 (9.9%)
NHS Lothian	424 (20.2%)
NHS Tayside	167 (8.0%)
Island boards (NHS Western Isles, Shetland and Orkney)	23 (1.1%)
Unknown	7 (0.3%)
Infant sex^a^	
Female	622 (29.7%)
Male	677 (32.3%)
Unknown	799 (38.1%)
SIMD (quintiles)	
1 (most deprived)	375 (17.9%)
2	398 (19.0%)
3	388 (18.5%)
4	451 (21.5%)
5 (least deprived)	479 (22.8%)
Unknown	7 (0.3%)
Ethnicity	
African, Caribbean or Black	22 (1.1%)
Asian, Asian Scottish or Asian British	32 (1.5%)
Mixed/multiple ethnic groups, and other ethnic group	27 (1.3%)
White	793 (37.8%)
Unknown (including refused)	1224 (58.3%)

Pregnancy outcomes and maternal and infant characteristics of singleton pregnancies where the baby is diagnosed with Down's syndrome (DS) from the Scottish Linked Congenital Conditions Dataset (SLiCCD) cohort (Scotland, 2000–2021). Data is presented by the number of singleton pregnancies of a baby diagnosed with DS in each group, and as a percentage of the total number of singleton pregnancies in this cohort (n, %).

NHS, National Health Service; SD, standard deviation; SIMD, Scottish Index of Multiple Deprivation.

a Infant sex is reported here for all pregnancy outcomes. Unknown sex corresponds to spontaneous foetal loss and termination of pregnancy outcomes only. Infant sex reported by each pregnancy outcome is in Appendix 1, Table S3.

Over half (54.1% (1135/2098)) of the pregnancies with DS resulted in a LB, followed by TOP (42.0% (881/2098)), stillbirth (3.7% (78/2098)) and spontaneous foetal loss (0.2% (4/2098)).

### TB prevalence of singleton DS pregnancies in Scotland (2000–2021)

#### Time trend

**Fig. 1:**
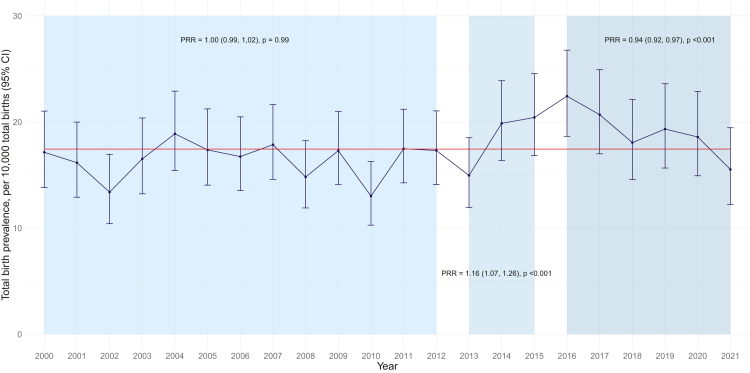
**Observed total birth prevalence of singleton pregnancies with Down's syndrome (2000**–**2021) by year of pregnancy end.** Total birth prevalence is presented as a blue line and 95% CIs are displayed as error bars for each year. The mean prevalence for the overall time period is presented as a red line. Piecewise regression prevalence rate ratios (PRRs), 95% CIs and p values are displayed for each time period analysed (2000–2012, 2013–2015, 2016–2021).

The TB prevalence of singleton pregnancies with DS for 2000–2021 was 17.4 (95% CI 16.7, 18.2) per 10,000 TBs in Scotland (yearly estimates are presented in Appendix 1, Table S4). The TB prevalence of DS varied from 13.0 (95% CI 10.3, 16.3) in 2010 to 22.4 (95% CI 18.7, 26.8) in 2016. The yearly change in TB prevalence of DS is presented in Fig. 1.

A non-linear trend in TB prevalence over time (2000–2021) was identified. Table 2 presents the regression results when assuming a linear time trend (2000–2021), and the piecewise regression results for the best fitting non-linear model. Details and outputs (including AIC values) from the full range of models explored are in Appendix 1, Table S5.

**Table 2:** Regression models estimates assuming linear and non-linear time trends (using piecewise regression) for total birth prevalence of Down's syndrome in Scotland between 2000 and 2021.

Model	Time period	Coefficient estimate (95% CI)	PRR (95% CI)	p value
Linear	2000–2021	0.009 (0.001, 0.017)	1.009 (1.001, 1.017)	0.029^a^
Piecewise regression output	Period 1 (2000–2012)	0.000 (−0.015, 0.015)	1.000 (0.995, 1.015)	0.990
	Period 2 (2013–2015)	0.149 (0.064, 0.234)	1.161 (1.066, 1.264)	<0.001^a^
	Period 3 (2016–2021)	−0.060 (−0.083, −0.036)	0.942 (0.920, 0.965)	<0.001^a^

Conway-Maxwell-Poisson (COM-Poisson) regression analysis was used to estimate the prevalence rate ratios (PRRs) and 95% confidence intervals (95% CI). Piecewise regression analysis estimates for the non-linear model presented. Spline estimates for other models explored are provided in Appendix 1, Table S5.

a Statistically significant.

**Fig. 2:**
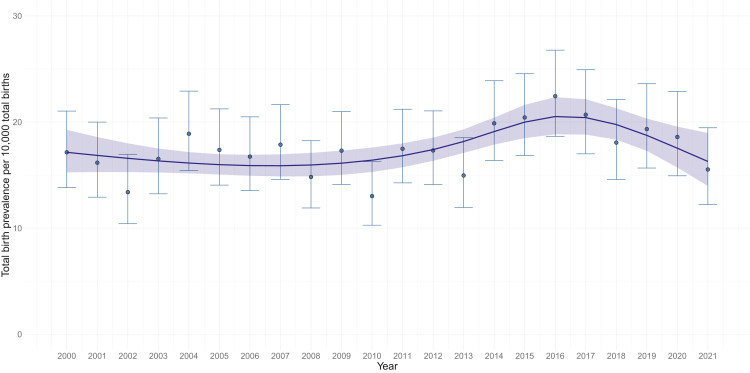
**Non-linear time trend estimates for total birth prevalence of singleton pregnancies with Down's syndrome in Scotland (year of pregnancy end).** Non-linear time trend (4 knots at 2000, 2013, 2016, 2021). Estimated model of mean total birth prevalence (blue line) and 95% CIs (blue ribbon) per year are plotted alongside the crude total birth prevalence for each year (blue dots and 95% CI error bar). The linear time trend model plot is in Appendix 1, Figure S2.

Piecewise regression estimates provide no evidence of a time trend between 2000 and 2012 (PRR 1.00, 95% CI 0.99, 1.02; p = 0.99), whereas there was an increasing trend in TB prevalence of DS between 2013 and 2015 (PRR 1.16, 95% CI 1.07, 1.26; p < 0.001) and a decreasing trend between 2016 and 2021 (PRR = 0.94, 95% CI 0.92, 0.97; p < 0.001) (Fig. 2, Table 2).

All subsequent analyses of TB prevalence adjusted for time trend have assumed this non-linear spline transformation to model time.

#### TB prevalence of DS and association with maternal socio-demographic factors

Table 3 reports the PRRs (95% CI) for the association between each socio-demographic factor and the TB prevalence of DS in unadjusted models, in a model adjusted for a non-linear time trend, and in a model adjusting for all socio-demographic factors. The crude TB and LB prevalence of DS by each socio-demographic factor are in Appendix 1, Table S6. VIFs for assessing multicollinearity between independent variables are reported in Appendix 1, Table S7.

**Table 3:** Regression model estimates for the association between total birth prevalence for Down's syndrome and maternal socio-demographic factors in Scotland between 2000 and 2021.

Coefficient	Unadjusted PRR (95% CI)	p value	Adjusted (time) PRR (95% CI)	p value	Adjusted (final) PRR (95% CI)	p value
**Maternal age**						
*Ref: <25*	*1*	*–*	*1*	*–*	*1*	*–*
25–29	1.08 (0.89, 1.31)	0.426	1.07 (0.88, 1.32)	0.467	1.07 (0.88, 1.3)	0.505
30–34	1.93 (1.63, 2.28)	<0.001^a^	1.93 (1.62, 2.29)	<0.001^a^	1.93 (1.63, 2.29)	<0.001^a^
35–39	5.36 (4.57, 6.28)	<0.001^a^	5.37 (4.53, 6.36)	<0.001^a^	5.37 (4.56, 6.36)	<0.001^a^
40+	13.48 (11.35, 16.01)	<0.001^a^	13.46 (11.25, 16.28)	<0.001^a^	13.59 (11.34, 16.22)	<0.001^a^
**Maternal SIMD**						
*Ref: 1 (most deprived)*	*1*	*–*	*1*	*–*	*1*	*–*
2	1.29 (1.12, 1.48)	<0.001^a^	1.28 (1.13, 1.46)	<0.001^a^	1.07 (0.92, 1.23)	0.359
3	1.39 (1.21, 1.61)	<0.001^a^	1.39 (1.22, 1.6)	<0.001^a^	0.98 (0.84, 1.14)	0.812
4	1.62 (1.41, 1.85)	<0.001^a^	1.62 (1.42, 1.84)	<0.001^a^	1 (0.86, 1.16)	0.999
5 (least deprived)	1.87 (1.63, 2.14)	<0.001^a^	1.88 (1.65, 2.12)	<0.001^a^	1.01 (0.87, 1.17)	0.893
**Maternal NHS health board of residence**						
*Ref: NHS Ayrshire and Arran*	*1*	*–*	*1*	*–*	*1*	*–*
NHS Borders	0.59 (0.37, 0.93)	0.022^a^	0.58 (0.36, 0.91)	0.019^a^	0.50 (0.32, 0.80)	0.004^a^
NHS Dumfries and Galloway	1.12 (0.82, 1.54)	0.482	1.12 (0.81, 1.54)	0.503	1.14 (0.83, 1.57)	0.421
NHS Fife	1.12 (0.88, 1.41)	0.357	1.12 (0.89, 1.4)	0.359	1.13 (0.89, 1.42)	0.329
NHS Forth Valley	1.01 (0.79, 1.3)	0.917	1.01 (0.79, 1.31)	0.919	0.94 (0.73, 1.22)	0.655
NHS Grampian	1.32 (1.07, 1.62)	0.009^a^	1.31 (1.06, 1.62)	0.010^a^	1.22 (0.98, 1.51)	0.073
NHS Greater Glasgow and Clyde	0.83 (0.68, 1.01)	0.066	0.83 (0.68, 1.01)	0.068	0.75 (0.61, 0.91)	0.005^a^
NHS Highland	1.33 (1.05, 1.69)	0.018^a^	1.34 (1.05, 1.68)	0.019^a^	1.2 (0.93, 1.52)	0.152
NHS Island Boards	0.98 (0.63, 1.51)	0.913	0.96 (0.61, 1.49)	0.849	0.83 (0.53, 1.3)	0.404
NHS Lanarkshire	0.8 (0.64, 0.99)	0.046^a^	0.8 (0.64, 0.99)	0.049^a^	0.77 (0.62, 0.96)	0.022^a^
NHS Lothian	1.28 (1.05, 1.55)	0.015^a^	1.27 (1.05, 1.55)	0.016^a^	1.06 (0.87, 1.3)	0.579
NHS Tayside	1.15 (0.92, 1.45)	0.229	1.15 (0.91, 1.45)	0.233	1.13 (0.9, 1.42)	0.316

Unadjusted, univariable Poisson regression model; Adjusted (time), COM-Poisson regression model adjusted for year (non-linear trend); Adjusted (final), COM-Poisson regression model adjusted for year (non-linear trend) and all factors; PRR, prevalence rate ratio; SIMD, Scottish index of multiple deprivation. Maternal age at conception. 95% CI, 95% confidence interval.

a Statistically significant.

##### Maternal age

**Fig. 3:**
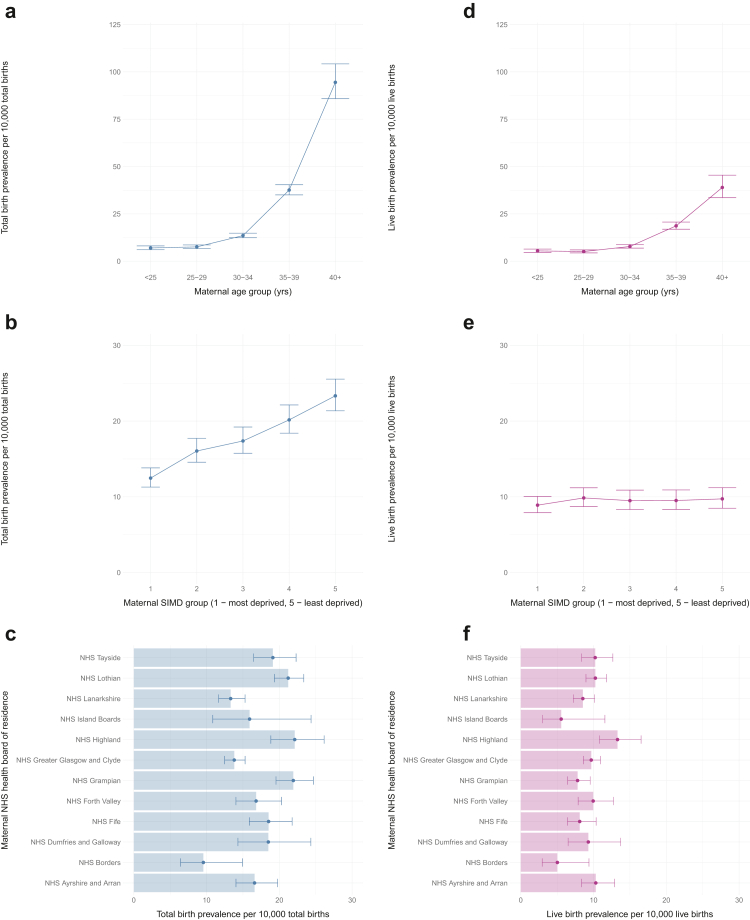
**Observed total birth (a–c) and live birth (d–f) prevalences (per 10,000 total or live births) of singleton Down's syndrome pregnancies in Scotland between 2000–2021 with 95% confidence intervals by maternal age group at conception (a, d), maternal SIMD group (b, e), and maternal NHS health board of residence (c, f).** SIMD, Scottish Index of Multiple Deprivation; SIMD 1, most deprived; SIMD 5, least deprived; Year, year of pregnancy end (a–c) or year of birth (d–f).

**Fig. 4:**
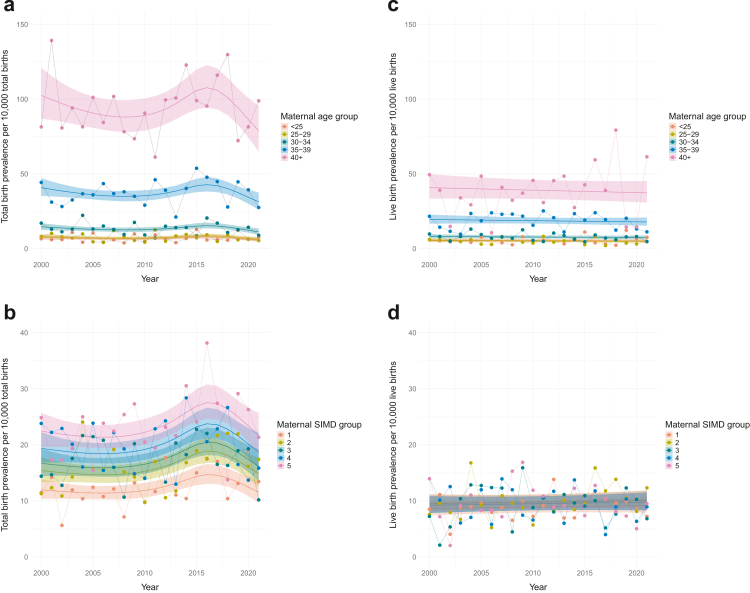
**Observed and predicted total birth (a, b) and live birth (c, d) prevalence of singleton pregnancies with Down's syndrome by maternal age group (a, c), and maternal SIMD group (b, d) over time (2000–2021).** Observed total birth prevalences marked by dots and model estimated total birth prevalence, adjusted by non-linear time trend shown as solid line and 95% CI ribbon. SIMD, Scottish Index of Multiple Deprivation; SIMD 1, most deprived; SIMD 5, least deprived; Year, year of pregnancy end.

The overall and yearly (2000–2021) observed TB prevalence of DS per maternal age group, and 95% CIs, were plotted (Figs. 3a and 4a, respectively).

When compared to mothers <25 years, those ≥30 years had a significantly increased TB prevalence of DS. Unadjusted prevalence in women aged 30–34 was 1.9 times (95% CI 1.63, 2.28, p < 0.001) that of women <25 years old, with women aged 35–39 and over 40 having 5.4 times (95% CI 4.57, 6.28, p < 0.001) and 13.5 times (95% CI 11.36, 16.01, p < 0.001) the prevalence of women <25 years, respectively (Table 3). Fig. 4a shows TB prevalence by maternal age group over time. The significance and effect size of these PRRs remained similar after adjusting for time and other factors (Table 3).

##### Scottish Index of Multiple Deprivation (SIMD)

The observed overall and yearly TB prevalences for each SIMD group (and 95% CIs) are presented in Figs. 3b and 4b, showing an increase in TB prevalence with increasing SIMD. Compared to those living in the most deprived areas (SIMD 1), mothers from SIMD areas 2–5 all had a significantly higher unadjusted TB prevalence rate for DS (Table 3), the least deprived areas (SIMD 5) had almost two times (PRR 1.87, 95% CI 1.63, 2.14; p < 0.001) the TB prevalence of DS of mothers in SIMD 1. Although the association with SIMD groups remained significant when adjusted for time trend, it lost significance when adjusted for other factors (Table 3). This could be explained by the highly significant linear relationship (χ^2^ = 130.73, p < 0.001) between SIMD and maternal age, with maternal age increasing as SIMD increased: SIMD 1 and SIMD 2 have more younger mothers with a pregnancy with DS (18%, 15%, respectively) compared to SIMD 4 and SIMD 5 (5% and 2%, respectively) (Appendix 1, Table S8).

##### Maternal NHS health board of residence

The TB prevalence of DS varied by NHS health board of residence (Fig. 3c). Compared to the reference (NHS A&A), NHS Borders had 0.6 times (95% CI 0.37, 0.93, p = 0.022) and NHS Lanarkshire 0.8 times (95% CI 0.64, 1.0, p = 0.046) its TB prevalence. In contrast, NHS Grampian, NHS Highland and NHS Lothian had significantly higher TB prevalences (Table 3).

Although these associations remained when adjusted by a non-linear time trend, only the difference in NHS Borders and NHS Lanarkshire remained statistically significant in the final adjusted model, and NHS Greater Glasgow and Clyde had a new significantly lower prevalence (Table 3 and Appendix 1, Figure S3a).

### LB prevalence of singleton DS pregnancies in Scotland (2000–2021)

Of the 2098 singleton pregnancies with DS, 1135 resulted in a LB (Table 1).

#### Overall time trend for LB prevalence of DS

**Fig. 5:**
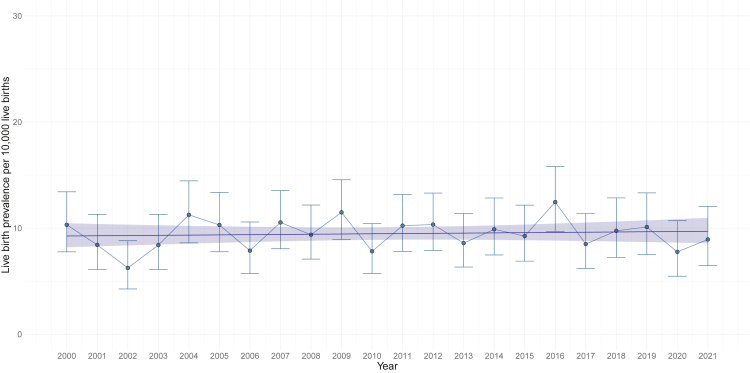
**Observed and estimated live birth prevalence of singleton pregnancies with Down's syndrome assuming linear time trend (2000**–**2021).** Estimated model of mean live birth prevalence (blue line) and 95% CIs (blue ribbon) per year plotted alongside the observed live birth prevalence for each year (blue points and 95% confidence intervals). Year, year of birth.

Fig. 5 shows the crude LB prevalence of DS in Scotland per year, between 2000 and 2021. The overall LB prevalence was 9.48 (95% CI 8.93, 10.04) per 10,000 LBs, remaining stable over this time.

Table 4 summarises the key regression model estimates investigating the time trend in LB prevalence of DS (linear and non-linear). Appendix 1, Table S9 presents all time trend models investigated for LB prevalence of DS.

**Table 4:** Regression model estimates assuming linear and non-linear time-trends for live birth prevalence of Down's syndrome in Scotland between 2000 and 2021.

Time-trend	Time period	Coefficient estimate (95% CI)	PRR (95% CI)	p value
Linear	2000–2021	0.00 (−0.01, 0.01)	1.00 (0.99, 1.01)	0.663
Non-linear (3 knots^a^)	Spline 1 (2000–2011)	0.02 (−0.01, 0.04)	–	0.204
	Spline 2 (2012–2021)	−0.02 (−0.05, 0.01)	–	0.232

PRR, prevalence rate ratio; 95% CI, 95% Confidence Interval.

a Equally spaced over time.

There is no evidence of a linear or non-linear time trend in the LB prevalence of pregnancies with DS in Scotland between 2000 and 2021 (p > 0.05) (Table 4 and Fig. 5).

#### LB prevalence of DS and association with maternal socio-demographic factors, and infant sex

Table 5 shows the association between each socio-demographic factor and the LB prevalence of DS in unadjusted models, in a model adjusted for time trend, and in a model adjusting for all socio-demographic factors.

**Table 5:** Regression model estimates for the association between live birth prevalence for Down's syndrome and maternal socio-demographic factors in Scotland between 2000 and 2021.

Coefficient	Unadjusted PRR (95% CI)	p value	Adjusted (time) PRR (95% CI)	p value	Adjusted (final) PRR (95% CI)	p value
**Maternal age**						
*Reference: <25*	*1*	*–*	*1*	*–*	*–*	*–*
25–29	0.94 (0.75, 1.17)	0.572	0.94 (0.74, 1.20)	0.626	0.98 (0.78, 1.22)	0.832
30–34	1.43 (1.17, 1.74)	<0.001^a^	1.43 (1.16, 1.79)	0.001^a^	1.57 (1.27, 1.93)	<0.001^a^
35–39	3.42 (2.83, 4.14)	<0.001^a^	3.46 (2.80, 4.22)	<0.001^a^	3.82 (3.13, 4.66)	<0.001^a^
40+	7.15 (5.73, 8.93)	<0.001^a^	7.24 (5.70, 9.12)	<0.001^a^	7.92 (6.3, 9.97)	<0.001^a^
**SIMD**						
*Reference: 1*	*1*	*–*	*1*	*–*	*–*	*–*
2	1.11 (0.93, 1.32)	0.250	1.11 (0.93, 1.31)	0.239	1.01 (0.84, 1.21)	0.920
3	1.07 (0.89, 1.28)	0.480	1.06 (0.90, 1.27)	0.470	0.87 (0.72, 1.05)	0.157
4	1.07 (0.89, 1.28)	0.466	1.07 (0.90, 1.27)	0.458	0.79 (0.66, 0.97)	0.021^a^
5	1.09 (0.91, 1.31)	0.337	1.09 (0.91, 1.31)	0.324	0.73 (0.60, 0.89)	0.002^a^
**Maternal NHS health board of residence**						
*Reference: NHS Ayrshire and Arran*	*1*	*–*	*1*	*–*	*–*	*–*
NHS Borders	0.51 (0.27, 0.94)	0.031^a^	0.49 (0.26, 0.90)	0.024^a^	0.46 (0.25, 0.87)	0.017^a^
NHS Dumfries and Galloway	0.91 (0.59, 1.40)	0.673	0.9 (0.59, 1.38)	0.628	0.93 (0.6, 1.45)	0.746
NHS Fife	0.79 (0.57, 1.09)	0.144	0.79 (0.57, 1.07)	0.132	0.81 (0.59, 1.13)	0.212
NHS Forth Valley	0.97 (0.70, 1.34)	0.836	0.96 (0.7, 1.32)	0.824	0.94 (0.68, 1.31)	0.736
NHS Grampian	0.76 (0.56, 1.01)	0.061	0.76 (0.57, 1.01)	0.054	0.78 (0.58, 1.05)	0.105
NHS Greater Glasgow and Clyde	0.94 (0.73, 1.20)	0.598	0.94 (0.73, 1.20)	0.609	0.87 (0.68, 1.12)	0.284
NHS Highland	1.29 (0.95, 1.75)	0.101	1.28 (0.96, 1.73)	0.092	1.22 (0.9, 1.67)	0.208
NHS Island Boards	0.57 (0.28, 1.15)	0.118	0.54 (0.26, 1.11)	0.092	0.5 (0.24, 1.03)	0.061
NHS Lanarkshire	0.83 (0.63, 1.09)	0.172	0.83 (0.63, 1.08)	0.165	0.8 (0.61, 1.06)	0.130
NHS Lothian	0.99 (0.77, 1.28)	0.942	0.99 (0.77, 1.27)	0.957	0.91 (0.7, 1.19)	0.496
NHS Tayside	0.99 (0.73, 1.34)	0.955	0.99 (0.74, 1.32)	0.954	1.01 (0.74, 1.36)	0.962
**Infant sex**						
*Reference: female*	*1*	*–*	*1*	*–*	*–*	*–*
Male	1.04 (0.93, 1.17)	0.485	1.04 (0.92, 1.17)	0.502	1.05 (0.93, 1.19)	0.427
**Year (linear time-trend)**						
Year (2000–2021)	1 (0.99, 1.01)	0.663	–	–	–	–

PRR, prevalence rate ratio; 95% CI, 95% Confidence Interval; Unadjusted, univariable COM-Poisson regression model; Adjusted (time), COM-Poisson regression model adjusted for year (linear trend); Adjusted (final), COM-Poisson regression model adjusted for all factors.

a Statistically significant.

##### Maternal age

The overall and yearly LB prevalences of DS per maternal age group are shown in Figs. 3d and 4c, and Appendix 1, Table S6. The LB prevalence increased with maternal age, from 5.44 (95% CI 4.6, 6.4) to 38.94 (95% CI 33.26, 45.32) per 10,000 LBs in those <25 years and ≥40 years, respectively.

Mothers older than 30 years old had significantly higher LB prevalence of DS compared to the reference (<25 years). The 30–34 group had 1.43 times (95% CI 1.17, 1.74, p < 0.001), the 35–39 year group 3.42 times (95% CI = 2.83, 4.14, p < 0.001) and the ≥40 age group over seven times (95% CI 5.73, 8.93, p < 0.001) the LB prevalence of women <25 years of age (Table 5). The significance and effect size of these associations remained similar after adjusting for time and all other factors (Table 5).

##### Scottish Index of Multiple Deprivation (SIMD)

The observed LB prevalences of DS by each SIMD group are shown in Fig. 3e, with little variation (∼10 per 10,000 LBs).

The regression results confirm that there was no evidence of an association between SIMD group and LB prevalence, either unadjusted or when adjusted for time (Table 5, Fig. 4d). However, there was evidence in the final model that SIMD groups 4 and 5 had a significantly lower prevalence compared to SIMD 1, with PRRs of 0.79 (95% CI 0.66, 0.97) and 0.73 (95% CI 0.60, 0.89), respectively.

#### Maternal NHS health board of residence

The observed overall and yearly LB prevalences of DS per maternal NHS health board of residence are shown in Fig. 3f and Appendix 1 Figure S3b. Variation is observed, with a lower LB prevalence in NHS Borders (5.03 (95% CI 2.51, 9.0)) and NHS Island Boards (5.55 (95% CI 2.4, 10.95)) and higher LB prevalence in NHS Highland (13.3 (95% CI 10.62, 16.44)) compared with NHS A&A (10.31 (95% CI 8.19, 12.82)).

The unadjusted model outputs (Table 5) show NHS Borders had a 0.51 times lower LB prevalence on average, compared to the reference group (95% CI 0.27, 0.94, p = 0.03). No other health board had a statistically significant difference in LB prevalence compared to NHS A&A, and there was no change after adjusting for time trend or in the final adjusted model (Table 5).

##### Infant sex

The LB prevalence of DS was not significantly different between males (9.67; 95% CI 8.91, 10.48) and females (9.27; 95% CI 8.51, 10.09) (Table 5, Appendix 1 Figure S4).

### Validation of SLiCCD against eSLiCCD

Between April 2019 and December 2020, 84 of the 87 pregnancies recorded in SLiCCD with DS were confirmed with DS in eSLiCCD, giving a PPV of 97% [95% CI 90, 99] (Appendix 1, Table S10). Three pregnancies with DS in SLiCCD were not confirmed in eSLiCCD. Of the 127 pregnancies with DS recorded in eSLiCCD, 84 were matched to a record in SLiCCD, giving a sensitivity of 66% [95% CI 57, 74].

None of the 38 pregnancies with DS in eSLiCCD but not in SLiCCD were present in SLiCCD under a different diagnosis. The PPV of SLiCCD was similar between pregnancy outcomes, whilst the sensitivity was lower for stillbirths (Appendix 1, Table S11).

## Discussion

This population-based study of singleton pregnancies with DS found evidence of an increase in TB prevalence of DS in Scotland between 2013 and 2015, followed by a decrease from 2016 onwards. In contrast, there was no evidence of a change in the LB prevalence of DS over time. Our reported prevalences of DS in Scotland (2000–2021) of 17.43 (95% CI 16.69, 18.19) per 10,000 TBs and 9.48 (95% CI 8.93, 10.04) per 10,000 LBs, respectively, are consistent with other reports on DS prevalence in Scotland.^15^^,^^16^ Unlike previous reports and studies,^14^^,^^15^ we have also analysed the association of TB and LB prevalence of DS in Scotland with socio-demographic factors, using robust and consistent denominator data.

Maternal age was significantly associated with both TB and LB prevalence of DS, even after adjustment for other factors; those aged 40+ had 13.5 times (95% CI 11.6, 16.01) the TB prevalence of those <25 years, and 7.15 times (95% CI 5.73, 8.93) their LB prevalence. These results align with established knowledge on the association between maternal age and the likelihood of a pregnancy with DS.^26^^,^^27^ The association with maternal age in our results is stronger for TB prevalence than LB prevalence. Lifestyle changes and fertility care service expansion have led to increasing pregnancy rates in those of advanced maternal age. A study investigating LBs and antenatal diagnoses of babies with DS in England and Wales between 1989 and 2008 found no increase in the number of LBs despite a major increase in the maternal age.^28^ They suggested this was due to increased screening and antenatal diagnostic testing in those under 37 years old, and a consistent number of women with an antenatal diagnosis of DS who chose to terminate their pregnancy. This is consistent with our results which show LB prevalence of DS is lower than TB prevalence in those over 35 years old.

There was also evidence of an association between TB and LB prevalence of DS and maternal NHS health board of residence, which persisted in the adjusted models. This could represent population-level or infrastructural differences that we were unable to adjust for. Maternal SIMD group was also associated with TB prevalence of DS and remained so after adjustment by time trend but not after adjustment for all factors. Further assessment found evidence of a linear relationship between SIMD and maternal age group, which may explain the loss of significance; an increase in SIMD group was matched by a linear increase in average maternal age. There was no evidence of an association between infant sex and LB prevalence of DS, consistent with research suggesting that, while some congenital conditions, such as genital and digestive disorders, show a greater prevalence in males, this difference does not exist for DS.^29^

This study has several strengths. It provides real-world estimates of TB and LB prevalence of DS in Scotland and associations with socio-demographic factors, using robust statistical methods. The dataset had minimal missing data for key variables such as maternal age, SIMD, and health board of residence, and SLiCCD remains the most comprehensive source for this cohort in Scotland from 2000 to 2021. Our findings align with other DS prevalence reports and official statistics from PHS.^15^

Uniquely, this is the first Scottish study to examine associations between socio-demographic factors and both TB and LB prevalence of DS. We used a consistent denominator and followed EUROCAT conventions to enable comparison with other populations. The inclusion of detailed annual breakdowns supports long-term monitoring of trends and the impact of screening changes in Scotland.

This study also has limitations. We could not adjust for all potential factors associated with DS prevalence. Ethnicity data, for example, were missing in nearly 60% of cases and were not routinely collected before 2003. We also limited non-linear regression models to four knots to avoid overfitting.

Only singleton pregnancies were included, ensuring comparability over time in screening practices, as there were substantial changes in screening for twin pregnancies with the 2020 introduction of NIPT. However, with only one year of post-implementation data, we were limited in assessing the impact of NIPT.

Finally, we categorised maternal age rather than modelling it as a continuous variable, which may reduce statistical precision. However, age categorisation offers greater clinical utility in public health research, facilitating the identification of linear or non-linear trends as age increases, as well as the allocation of resources and targeting of interventions to specific groups, which would be difficult if guided only by an estimate of yearly increase in prevalence.

The time trends in TB versus LB prevalence of DS in our cohort differed which likely reflects a combination of factors. One is a genuine change in the underlying prevalence of DS, potentially due to shifts in the conception population with increasing maternal age over time. Another contributing factor may be improvements in early screening and detection, including the transition to first-trimester screening and the implementation of NIPT in 2020.^11^^,^^30^

Differences in case ascertainment among pregnancy outcomes may also contribute to trends, due to variation in the completeness and quality of the data sources within SLiCCD. Notably, a change in how babies with congenital conditions were reported occurred in 2002, when neonatal discharges shifted from the SMR11 system to the Scottish Birth Record (SBR) system.^15^ This likely contributed to the decrease in both TB and LB prevalence observed in 2002.

In addition, there are known issues with the recording of diagnostic codes within SBR by some NHS boards which may affect the accurate identification of babies with congenital conditions, and potentially impact their inclusion in SLiCCD.^21^ Consequently, there may be under-ascertainment of babies with congenital conditions, including DS, in SLiCCD—affecting most NHS boards from 2017 onwards. This may underlie or contribute to the falling TB prevalence, and to a lesser extent the LB prevalence of DS seen in our data from 2017 onwards.

We analysed the ascertainment of SLiCCD by comparing it to eSLiCCD, a dataset enhanced with confirmatory genetic testing data (2019–2020). The high PPV of babies with DS in SLiCCD for all pregnancy outcomes suggests we can be confident that pregnancies in SLiCCD were correctly diagnosed with DS. However, the sensitivity of SLiCCD for DS is low, particularly for stillbirths, suggesting under-ascertainment of babies with DS, and underestimation of the true prevalence. Notably, the number of pregnancies included in these validation analyses, especially for stillbirths, was very small (n = 5 in eSLiCCD) meaning that small differences in observed numbers can have a disproportionate effect on estimates. After 2021, the Congenital Conditions and Rare Diseases Registration and Information Service for Scotland (CARDRISS) dataset replaced SLiCCD, using more robust methods for ascertainment of babies with congenital conditions.^21^

To the best of the authors’ knowledge, this study provides the most up-to-date and comprehensive analysis of the prevalence of singleton pregnancies with DS in Scotland between 2000 and 2021. Information on these trends is valuable for monitoring of this population alongside screening changes. We provide context for Scottish NIPT implementation and trends within socio-demographic groups. Our analysis builds on previous reports by providing a more in-depth assessment of long-term trends for singleton pregnancies with DS since 2000. We used a consistent NRS denominator source, providing breakdowns for the Scottish population per year. The prevalences reported in this study are therefore the best estimates currently available for the Scottish population, alongside maternal and infant socio-demographic data for the period 2000–2021. Ultimately, by providing prevalence estimates for DS across key socio-demographic groups, this study will help inform ongoing policy decisions around service provision and screening for DS. Future research should focus on how the screening tests offered, particularly the addition of NIPT, may influence pregnancy outcomes.

## Contributors

RV and RW were responsible for the supervision, administration, funding acquisition and resources for this project. ES, RV, and RW designed the study. ES and RV conducted the analyses (data curation, investigation, formal analysis, visualisation). All authors contributed to interpretation. RV validated the code and analyses. ES drafted the manuscript, and all authors reviewed and contributed to its writing. ES, RV and RW had access to the data. All authors are responsible for the decision of submitting the manuscript.

## Data sharing statement

Subject to governance approvals, researchers can access pseudonymised extracts of datasets used in this study through the Scottish NHS Safe Haven facility supported by Public Health Scotland. Interested researchers should submit an initial enquiry form to Research Data Scotland (https://www.researchdata.scot/accessing-data/).

## Declaration of interests

We declare no competing interests.
