# Supplementary material for: Total and live birth prevalence of singleton pregnancies with Down's syndrome in Scotland between 2000 and 2021: a population-based study
Source: Lancet Reg Health Eur. 2026 Mar 6;65:101639. doi: 10.1016/j.lanepe.2026.101639 (PMC12992100; doi:10.1016/j.lanepe.2026.101639)
Supplement: Supplementary File 1 [file mmc1.docx]

**Total and livebirth prevalence of singleton pregnancies with Down’s Syndrome in Scotland between 2000 and 2021: a population-based study**

**Appendix 1**

**Table of contents:**

National data sources used for the creation of Scottish Linked Congenital Conditions Dataset (SLiCCD) --------2

Figure S1: Map of Scotland divided by NHS health boards, including population estimates (2022). ---------------2

Table S1: 2022 population estimates per NHS health board in Scotland ------------------------------------------------2

Scottish Index of Multiple Deprivation (SIMD) ----------------------------------------------------------------------------3

Table S2: Baseline characteristics. Pregnancy outcomes and maternal and infant characteristics within the SLiCCD cohort of singleton pregnancies (Scotland, 2000-2019). --------------------------------------------------------3

Table S3: Infant sex by pregnancy outcome, within the Scottish Linked Congenital Conditions Dataset (SLiCCD), 2000-2021. -----------------------------------------------------------------------------------------------------------------------4

Table S4: Total and live birth prevalence of singleton pregnancies with Down’s syndrome per year. --------------4

Table S5: Summary of regression model outputs assuming a linear and a non-linear time trend (piecewise regression) for total birth prevalence of Down’s syndrome in Scotland between 2000-2021. ------------------------4

Figure S2: Total birth prevalence of Down’s syndrome (per 10,000 total births): linear time trend model for predicted total birth prevalence of babies with Down’s syndrome in Scotland over time (year of pregnancy end). --------------------------------------------------------------------------------------------------------------------------------------5

Table S6: Total and live birth prevalence per socio-demographic factors of interest. ----------------------------------5

Figure S3: Observed and predicted total and live birth prevalence of singleton pregnancies with Down’s syndrome in Scotland by maternal NHS health board of residence, over time (2000-2021). -------------------------------------6

Table S7: Summary of variance inflation factor analysis for final adjusted total and live birth prevalence models---------------------------------------------------------------------------------------------------------------------------------------6

Table S8: Age group distribution (percentage) within each SIMD group (by row), for singleton pregnancies with Down’s syndrome. --------------------------------------------------------------------------------------------------------------6

Figure S4: Observed live birth prevalence in SLiCCD cohort, by infant sex, over time. ------------------------------7

Table S9: Summary of regression model results (COM-Poisson) for investigating the presence of a linear time trend for live birth prevalence of Down’s syndrome in Scotland between 2000-2021.  -------------------------------7

Table S10: Validation of SLiCCD vs enhanced SLiCCD, for singleton pregnancies with Down’s syndrome. -----7

Table S11: Validation of SLiCCD vs enhanced SLiCCD, for babies with Down’s syndrome, by pregnancy outcome. --------------------------------------------------------------------------------------------------------------------------7

**National data sources used for the creation of Scottish Linked Congenital Conditions Dataset (SLiCCD)**

The following national data sources were used by Public Health Scotland to identify pregnancies for inclusion in SLiCCD, and provide additional clinical and socio-demographic information on pregnancies: ^1^

• National Records of Scotland (NRS) statutory live births, stillbirths, and deaths for babies up to 1 year.

• Hospital maternity care delivery, miscarriage, and termination of pregnancy discharge, Scottish Morbidity Records (SMR02)

• Statutory termination of pregnancy notifications (Abortion Act Scotland records [AAS])

• Hospital neonatal care discharge:

o SMR11 records from 2000 – April 2003

o Scottish Birth Record (SBR) from April 2003

• General hospital discharge records for babies aged up to 1 year (SMR01)

• Perinatal death enhanced surveillance:

o Scottish Stillbirth and Infant Death (SSBID) records for 2000 – 2012

o Mothers and Babies: Reducing Risk through Audits and Confidential Enquiries across the UK (MBRRACE-UK) records for 2013 – 2020

**Figure S1: Map of Scotland divided by NHS health boards, including population estimates (2022)**


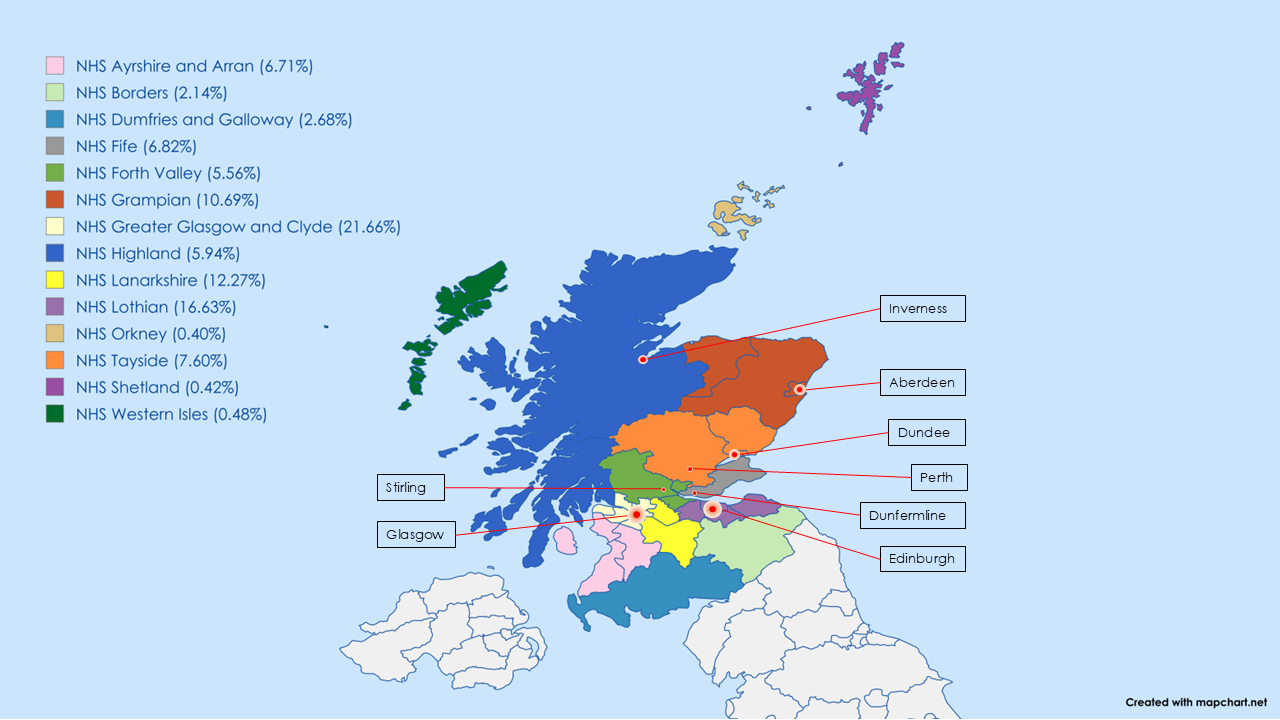


*Presenting the 14 NHS health boards in Scotland (map key), and eight cities (red dots and text labels). Total population estimate for 2022 = 5,447,700. Population estimates for each health board in 2022 have been displayed as a proportion of the total Scottish population, from data sourced from statistics.gov.scot.^2^ Map created using*[*Mapchart.net*](http://mapchart.net/)*under a CC BY 4.0 license.*

**Table S1: 2022 population estimates per NHS health board in Scotland**

| **Health Board Areas** | **Population** | **Percentage of total population (%)** |
| --- | --- | --- |
| Ayrshire and Arran | 365,440 | 6·71% |
| Borders | 116,820 | 2·14% |
| Dumfries and Galloway | 145,770 | 2·68% |
| Fife | 371,340 | 6·82% |
| Forth Valley | 302,730 | 5·56% |
| Grampian | 582,220 | 10·69% |
| Greater Glasgow and Clyde | 1,179,910 | 21·66% |
| Highland | 323,630 | 5·94% |
| Lanarkshire | 668,360 | 12·27% |
| Lothian | 906,190 | 16·63% |
| Orkney | 22,020 | 0·40% |
| Shetland | 23,020 | 0·42% |
| Tayside | 414,130 | 7·60% |
| Western Isles | 26,120 | 0·48% |
| Total | 5,447,700 | 100·00% |

*Population estimates for each health board in 2022 have been displayed as a proportion of the total Scottish population, from data sourced from statistics.gov.scot.*

**Scottish Index of Multiple Deprivation (SIMD)**

The Scottish Linked Congenital Conditions Dataset (SLiCCD) contains the maternal SIMD for each pregnancy. SIMD was first produced by the Scottish Government in 2004, and was updated in 2006, 2009, 2012, 2016 and 2020. The SIMD quintiles in SLiCCD are assigned by maternal postcode at end of pregnancy. Those ending 2000-2003 use SIMD 2004, 2004-2006 use SIMD 2006, 2007-2009 use SIMD 2009, 2010-2013 use SIMD 2012, 2014-2016 use SIMD 2016, and 2017 – 2021 use SIMD 2020.

**Table S2: Baseline characteristics. Pregnancy outcomes and maternal and infant characteristics within the SLiCCD cohort of singleton pregnancies (Scotland, 2000-2019).**

|  | **Singleton pregnancies with DS in Scotland between 2000 and 2019 (n=1934)** |
| --- | --- |
| **Pregnancy outcome** | |
| Live births | 1055 (54·55%) |
| Stillbirths | 77 (3·98%) |
| Late fetal loss | 4 (0·21%) |
| Termination of pregnancy | 798 (41·26%) |
| **Maternal age (grouped years, at conception)** | |
| <20 | 66 (3·41%) |
| 20-24 | 114 (5·89%) |
| 25-29 | 226 (11·69%) |
| 30-34 | 453 (23·42%) |
| 35-39 | 698 (36·09%) |
| 40+ | 370 (19·13%) |
| Unknown | 7 (0·36%) |
| **Maternal age (years, at conception)** | |
| Mean (SD) | 34·07 (6·34) |
| **NHS health board of residence** | |
| NHS Ayrshire and Arran | 122 (6·31%) |
| NHS Borders | 20 (1·03%) |
| NHS Dumfries and Galloway | 51 (2·64%) |
| NHS Fife | 142 (7·34%) |
| NHS Forth Valley | 102 (5·27%) |
| NHS Grampian | 265 (13·70%) |
| NHS Greater Glasgow and Clyde | 337 (17·43%) |
| NHS Highland | 132 (6·83%) |
| NHS Lanarkshire | 186 (9·62%) |
| NHS Lothian | 389 (20·11%) |
| NHS Tayside | 7 (0·36%) |
| Island boards (NHS Western Isles, Shetland and Orkney) | 10 (0·52%) |
| Unknown | 158 (8·17%) |
| **Infant sex*** | |
| Female | 582 (30·09%) |
| Male | 635 (32·83%) |
| Unknown | 717 (37·07%) |
| **SIMD (quintiles)** | |
| 1 (most deprived) | 338 (17·48%) |
| 2 | 365 (18·87%) |
| 3 | 362 (18·72%) |
| 4 | 422 (21·82%) |
| 5 (least deprived) | 440 (22·75%) |
| Unknown | 7 (0·36%) |
| **Ethnicity** | |
| African, Caribbean or Black | 18 (0·93%) |
| Asian, Asian Scottish or Asian British | 27 (1·40%) |
| Mixed/multiple ethnic groups, and other ethnic group | 16 (0·83%) |
| White | 684 (35·37%) |
| Unknown (including refused) | 1180 (61·01%) |

*Data presented as number of pregnancies with Down’s syndrome* *(% of total singleton pregnancies with Down’s syndrome). *Infant sex is reported here for all pregnancy outcomes. Unknown sex corresponds to late fetal loss and termination of pregnancy outcomes only. SD = standard deviation, SIMD = Scottish Index of Multiple Deprivation.*

**Table S3: Infant sex by pregnancy outcome, within the Scottish Linked Congenital Conditions Dataset (SLiCCD), 2000-2021.**

| **Pregnancy outcome** | **Male** | **Female** | **Unknown** |
| --- | --- | --- | --- |
| Live birth | 594 | 541 | 0 |
| Stillbirth and Late Fetal Death | 41 | 40 | 1 |
| Termination of pregnancy | 42 | 41 | 798 |

**Table S4: Total and live birth prevalence of singleton pregnancies with Down’s syndrome per year.**

| **Year (end of pregnancy)** | **Total birth prevalence, per 10,000 total births (95% CI)** | **Live birth prevalence, per 10,000 live births (95% CI)** |
| --- | --- | --- |
| 2000 | 17·15 (13·83, 21·03) | 10·31 (7·77, 13·42) |
| 2001 | 16·17 (12·91, 19·99) | 8·42 (6·12, 11·30) |
| 2002 | 13·39 (10·42, 16·95) | 6·24 (4·27, 8·82) |
| 2003 | 16·53 (13·24, 20·38) | 8·40 (6·11, 11·28) |
| 2004 | 18·89 (15·42, 22·91) | 11·25 (8·61, 14·46) |
| 2005 | 17·37 (14·06, 21·24) | 10·30 (7·78, 13·37) |
| 2006 | 16·74 (13·53, 20·49) | 7·88 (5·73, 10·58) |
| 2007 | 17·87 (14·60, 21·65) | 10·54 (8·06, 13·54) |
| 2008 | 14·82 (11·90, 18·24) | 9·38 (7·08, 12·18) |
| 2009 | 17·30 (14·12, 20·98) | 11·48 (8·92, 14·56) |
| 2010 | 13·02 (10·28, 16·27) | 7·82 (5·72, 10·43) |
| 2011 | 17·48 (14·27, 21·20) | 10·23 (7·81, 13·17) |
| 2012 | 17·32 (14·11, 21·05) | 10·34 (7·89, 13·31) |
| 2013 | 14·96 (11·94, 18·53) | 8·59 (6·33, 11·38) |
| 2014 | 19·88 (16·38, 23·90) | 9·89 (7·47, 12·84) |
| 2015 | 20·43 (16·84, 24·56) | 9·26 (6·89, 12·17) |
| 2016 | 22·44 (18·65, 26·77) | 12·46 (9·67, 15·79) |
| 2017 | 20·69 (17·00, 24·94) | 8·50 (6·20, 11·37) |
| 2018 | 18·06 (14·58, 22·13) | 9·75 (7·23, 12·85) |
| 2019 | 19·34 (15·66, 23·61) | 10·11 (7·50, 13·32) |
| 2020 | 18·59 (14·93, 22·88) | 7·76 (5·46, 10·70) |
| 2021 | 15·53 (12·21, 19·46) | 8·94 (6·47, 12·04) |

*CI = confidence interval.*

**Table S5: Summary of regression model outputs assuming a linear and a non-linear time trend (with restricted cubic splines) for total birth prevalence of Down’s syndrome** **in Scotland between 2000-2021.**

| **Model type​** | **Coefficient ​** | **Estimate (95% CI)​** | **pRR (95% CI)** | **P value​** | **AIC​** |
| --- | --- | --- | --- | --- | --- |
| COM-Poisson​ (linear) | Year | 0·009 (0·001, 0·017)​ | 1·009 (1·001, 1·017) | 0·029*​ | 175 |
| Com-Poisson (non-linear, 3 knots)​ | Spline 1 | 0·006 (-0·014, 0·026)​ | - | 0·577​ | 177 |
|  | Spline 2 | 0·004 (-0·020, 0·028)​ | - | 0·724​ |  |
| COM-Poisson (non-linear, 4 knots)​ | Spline 1 | -0·011 (-0·041, 0·019)​ | - | 0·488​ | 177 |
|  | Spline 2 | 0·106 (-0·036, 0·248)​ | - | 0·142​ |  |
|  | Spline 3 | -0·214 (-0·501, 0·073)​ | - | 0·144​ |  |
| COM-Poisson (non-linear, 4 knots specified at 0, 10, 16, 21)​ | Spline 1(2000-2010) | -0·020 (-0·049, 0·008)​ | - | 0·162 | 173 |
|  | Spline 2 (2011-2015) | 0·074 (0·015, 0·133)​ | - | 0·013*​ |  |
|  | Spline 3 (2016-2021) | -0·440 (-0·764, -0·115)​ | - | 0·008*​ |  |
| Com-Poisson (non-linear, 4 knots specified at 2000, 2013, 2016, 2021)​ | Spline 1(2000-2012) | -0·017 (-0·040, 0·006) | - | 0·144​ | 170​ |
|  | Spline 2 (2013-2015) | 0·057 (0·018, 0·095)​ | - | 0·004*​ |  |
|  | Spline 3 (2016-2021) | -1·145 (-1·823, -0·466)​ | - | <0·001* |  |

*The best fitting model (lowest AIC) is 4 knots non-linear, specified at time points 2000, 2013, 2016 and 2021. Only linear and best non-linear model piecewise outputs are displayed in article results. *p<0.05. AIC = Akaike Information Criterion, CI = confidence interval, COM-Poisson = Conway-Maxwell Poisson.*

**Figure S2: Total birth prevalence of Down’s syndrome** **(per 10,000 total births): linear time trend model for predicted total birth prevalence of babies with Down’s syndrome** **in Scotland over time (year of pregnancy end).**


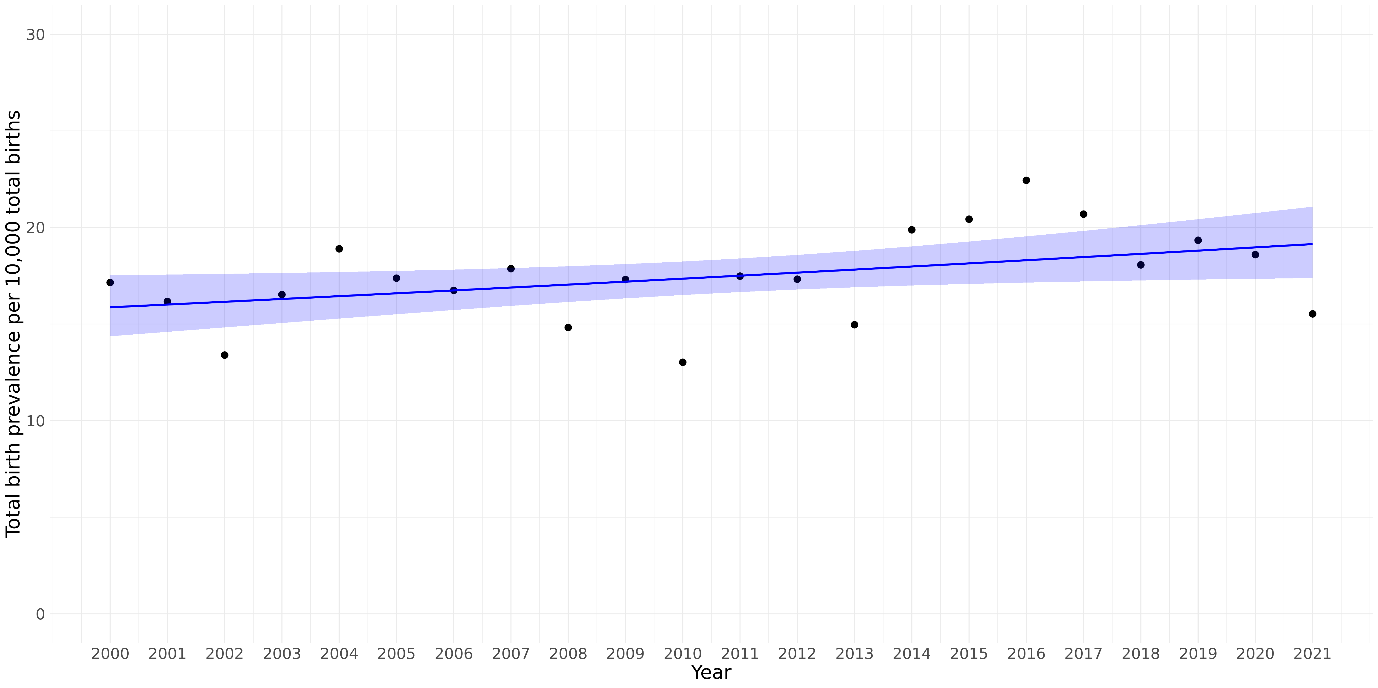


*Estimated model of mean total birth prevalence (blue line) and CIs (blue ribbon) per year are plotted alongside the crude total birth prevalence for each year (black points). Year = year of birth. CI = confidence interval.*

**Table S6: Total and live birth prevalence per socio-demographic factors of interest.**

| **Socio-demographic factor** | **Total birth prevalence of DS, per 10,000 total births (95% CI)** | **Live birth prevalence of DS, per 10,000 live births (95% CI)** |
| --- | --- | --- |
| Maternal age | | |
| <25 | 7·00 (6·04,8·06) | 5·44 (4·60, 6·4) |
| 25-29 | 7·56 (6·64,8·58) | 5·10 (4·35, 5·95) |
| 30-34 | 13·53 (12·36,14·78) | 7·77 (6·89, 8·74) |
| 35-39 | 37·58 (34·96,40·35) | 18·66 (16·82, 20·65) |
| 40+ | 94·47 (85·52,104·1) | 38·94 (33·26, 45·32) |
| SIMD quintile | | |
| 1 | 12·47 (11·24,13·8) | 8·90 (7·86,10·04) |
| 2 | 16·05 (14·51,17·71) | 9·85 (8·65,11·17) |
| 3 | 17·38 (15·69,19·19) | 9·49 (8·26,10·86) |
| 4 | 20·16 (18·34,22·11) | 9·51 (8·27,10·88) |
| 5 | 23·33 (21·29,25·52) | 9·73 (8·42,11·18) |
| NHS health board of residence | | |
| NHS Ayrshire and Arran | 16·59 (13·87,19·69) | 10·31 (8·19,12·82) |
| NHS Borders | 9·56 (5·92,14·61) | 5·03 (2·51, 9·00) |
| NHS Dumfries and Galloway | 18·49 (13·89,24·12) | 9·28 (6·12,13·51) |
| NHS Fife | 18·52 (15·71,21·69) | 8·10 (6·28,10·29) |
| NHS Forth Valley | 16·81 (13·84,20·22) | 9·95 (7·70,12·66) |
| NHS Grampian | 21·92 (19·43,24·63) | 7·81 (6·35,9·50) |
| NHS Greater Glasgow and Clyde | 13·80 (12·44,15·28) | 9·69 (8·55,10·93) |
| NHS Highland | 22·11 (18·63,26·06) | 13·3 (10·62,16·44) |
| NHS Island Boards | 15·91 (10·09,23·87) | 5·55 (2·40,10·95) |
| NHS Lanarkshire | 13·31 (11·56,15·25) | 8·53 (7·14,10·11) |
| NHS Lothian | 21·21 (19·24,23·33) | 10·25 (8·89,11·76) |
| NHS Tayside | 19·11 (16·32,22·24) | 10·23 (8·21,12·59) |
| Infant sex | | |
| Male | - | 9·67 (8·91,10·48) |
| Female | - | 9·27 (8·51,10·09) |

*DS = Down’s syndrome, SIMD = Scottish Index of Multiple Deprivation, CI = confidence interval.*

**Figure S3: Observed and predicted total (a) and live birth (b) prevalence of singleton pregnancies with Down’s syndrome** **in Scotland by maternal NHS health board of residence, over time (2000-2021).**


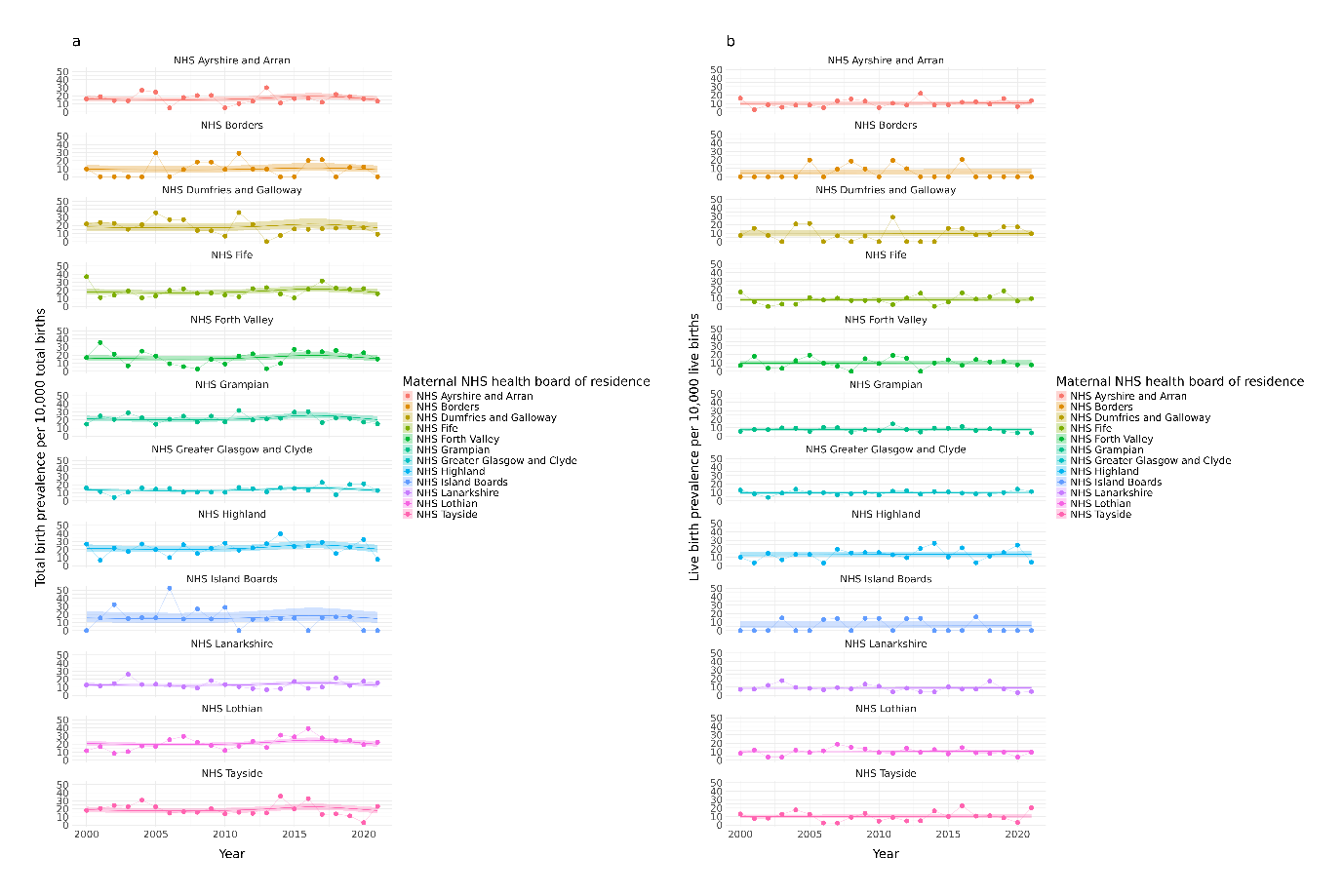
*Year = year of pregnancy end (a) and year of birth (b). Observed prevalences marked by dots and model estimated total birth prevalence of DS between maternal NHS health board of residence in SLiCCD cohort shown as solid line and 95% CI ribbon. CI = confidence interval.*

**Table S7: Summary of variance inflation factor analysis for final adjusted total and live birth prevalence models.**

| **Model** | **Term** | **VIF (95% CI)** |
| --- | --- | --- |
| Total birth prevalence (final adjusted model) | Age | 1·08 (1·06, 1·12) |
|  | SIMD | 1·23 (1·20, 1·27) |
|  | Health board of residence | 1·16 (1·13, 1·20) |
|  | Time period (non-linear) | 1·01 (1·00, 1·10) |
| Live birth prevalence (final adjusted model) | Age | 1·09 (1·08, 1·12) |
|  | Health board of residence | 1·16 (1·14, 1·18) |
|  | Sex | 1·00 (1·00, inf) |
|  | SIMD | 1·24 (1·22, 1·27) |
|  | Time period (linear) | 1·01 (1·00, 1·06) |

*VIF = variance inflation factor, CI = confidence interval, SIMD = Scottish Index of Multiple Deprivation.*

**Table S8: Age group distribution (percentage) within each SIMD group (by row), for singleton pregnancies with Down’s syndrome.**

|  | **Maternal age** | | | | | |
| --- | --- | --- | --- | --- | --- | --- |
| **SIMD** | **<25** | **25-29** | **30-34** | **35-39** | **40+** | **Total** |
| **1** | 18·23% | 15·28% | 20·11% | 28·15% | 18·23% | **100%** |
| **2** | 14·61% | 14·61% | 18·89% | 37·03% | 14·86% | **100%** |
| **3** | 8·79% | 9·56% | 26·36% | 34·63% | 20·67% | **100%** |
| **4** | 5·11% | 11·11% | 26·22% | 36·89% | 20·67% | **100%** |
| **5** | 1·46% | 7·72% | 25·26% | 43·22% | 22·34% | **100%** |

*SIMD = Scottish Index of Multiple Deprivation.*

**Figure S4: Observed live birth prevalence in SLiCCD cohort, by infant sex, over time.**


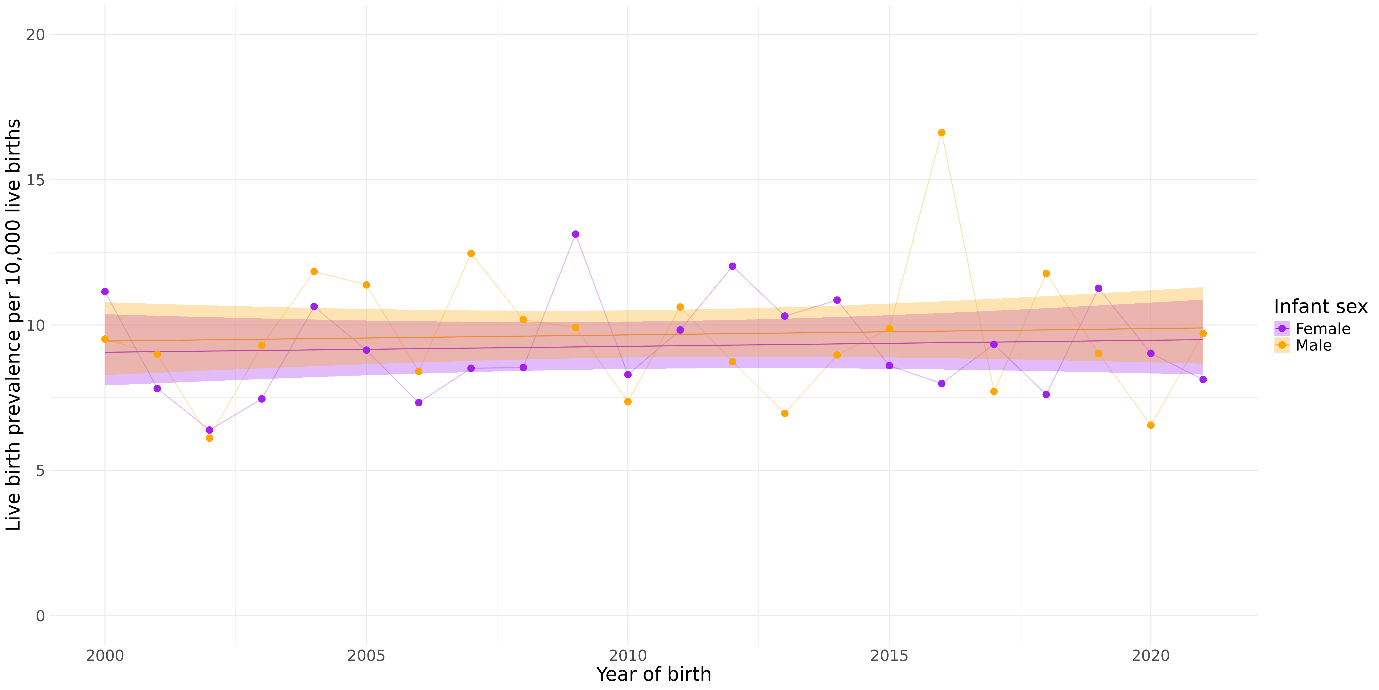


*Observed live birth prevalence plotted as points, with model estimated prevalence adjusted by linear time trend as line with 95% CI ribbon.*

**Table S9: Summary of regression model results (COM-Poisson) for investigating the presence of a linear time trend for live birth prevalence of Down’s syndrome** **in Scotland between 2000-2021.**

| **Model** | **Coefficient** | **Estimate (95%CI)** | **PRRs (95% CI)** | **P value** | **AIC** |
| --- | --- | --- | --- | --- | --- |
| COM-Poisson (linear) | Time | 0·002 (-0·01, 0·01) | 1 (0·99, 1·01) | 0·663 | 157·7 |
| COM-Poisson (non-linear) [3 knots] | Spline 2 | 0·02 (-0·01, 0·04) | 1·02 (0·99, 1·04) | 0·204 | 158·3 |
|  | Spline 3 | -0·02(-0·05, 0·01) | 0·98 (0·95, 1·01) | 0·232 |  |
| COM-Poisson (non-linear) [4 knots] | Spline 2 | 0·01 (-0·02, 0·05 | 1·01 (0·98, 1·05) | 0·468 | 160·3 |
|  | Spline 3 | -0·01(-0·19, 0·17) | 0·99 (0·83, 1·18) | 0·904 |  |
|  | Spline 4 | -0·004(-0·36, 0·36) | 1·00 (0·69, 1·43) | 0·985 |  |

*CI = confidence interval, COM-Poisson = Conway-Maxwell Poisson.*

**Table S10: Validation of SLiCCD vs enhanced SLiCCD, for singleton pregnancies with Down’s syndrome.**

|  | | **Enhanced SLiCCD (SLiCCD + genetic data)** | | | |
| --- | --- | --- | --- | --- | --- |
|  |  | Recorded as DS | Not recorded as DS (under other condition) | Not found in enhanced SLiCCD | Total |
| **SLiCCD** | Recorded as DS | 84 | 0 | 3 | 87 |
|  | Not recorded as DS (under other condition) | 0 |  |  |  |
|  | Not found in SLiCCD (genetics only record) | 38 |  |  |  |
|  | Total | 127 |  |  |  |

*Pregnancies ending in SLiCCD and eSLiCCD between 1^st^ April 2019 and 31^st^ December 2020, >20 weeks gestation only. DS = Down’s syndrome.*

**Table S11: Validation of SLiCCD vs enhanced SLiCCD, for babies with Down’s syndrome, by pregnancy outcome.**

| **Pregnancy outcome** | **Number of babies specified with DS in both SLiCCD and enhanced SLiCCD** | **Number of babies added to DS specified group in enhanced SLiCCD** | **Total number of babies in the DS specified group in enhanced SLiCCD** | **Total number of babies in the specified group in SLiCCD** | **Sensitivity of SLiCCD for DS** | **95% CI** | **PPV** | **95% CI** |
| --- | --- | --- | --- | --- | --- | --- | --- | --- |
| All outcomes | 84 | 38 | 127 | 87 | 0·66 | 0·57, 0·74 | 0·97 | 0·90, 0·99 |
| Live birth | 70 | 23 | 93 | 74 | 0·75 | 0·65, 0·84 | 0·95 | 0·87, 0·99 |
| Stillbirth or late fetal loss | 2 | 3 | 5 | 2 | 0·40 | 0·05, 0·85 | 1·00 | 0·16, 1·00 |
| Termination of pregnancy | 11 | 2 | 13 | 11 | 0·85 | 0·54, 0·98 | 1·00 | 0·71, 1·00 |

*CI = confidence interval, SIMD = Scottish Index of Multiple Deprivation, PPV = positive predictive value. DS = Down’s syndrome*

**References**

1. NHS National Services Scotland. *Congenital Anomalies in Scotland: 2012 to 2017* .; 2019. Accessed August 25, 2025. https://webarchive.nrscotland.gov.uk/20200215150610/https://www.isdscotland.org/Health-Topics/Maternity-and-Births/Publications/2019-11-26/2019-11-26-Congenital-Anomalies-in-Scotland-2017-Summary.pdf?3702944518

2. Scottish Government. Open access to Scotland’s official statistics. Statistics.gov.scot. Accessed January 26, 2026. https://statistics.gov.scot/home
